# Dose Optimization of Targeted Therapies for Oncologic Indications

**DOI:** 10.3390/cancers16122180

**Published:** 2024-06-09

**Authors:** Marjorie E. Zettler

**Affiliations:** Clinical Science, Accutar Biotechnology Inc., Cranbury, NJ 08512, USA; marjoriezettler@accutarbio.com

**Keywords:** dose optimization, dose-finding, drug development, targeted therapy, oncology drugs, cancer drugs

## Abstract

**Simple Summary:**

The majority of oncology drugs approved by the Food and Drug Administration over the past quarter century have been targeted therapies, designed to act on cancer cells with specific molecular abnormalities. Although these newer agents have different properties compared to cytotoxic chemotherapy, the same methodology used for dose-finding during drug development has continued to be used. Growing awareness of the inadequacy of traditional dose selection processes for modern cancer drugs has been the catalyst for the creation of new regulatory guidance aimed at reforming current practices. This review summarizes six cases, illustrating different approaches to targeted therapy dose optimization.

**Abstract:**

Therapeutic advances in oncology in the 21st century have contributed to significant declines in cancer mortality. Notably, targeted therapies comprised the largest proportion of oncology drugs approved by the United States (US) Food and Drug Administration (FDA) over the past 25 years and have become the standard of care for the treatment of many cancers. However, despite the metamorphosis of the therapeutic landscape, some aspects of cancer drug development have remained essentially unchanged. In particular, the dose-finding methodology originally developed for cytotoxic chemotherapy drugs continues to be implemented, even though this approach no longer represents the most appropriate strategy for modern cancer therapies. In recognition of the need to reconsider assumptions, adapt the dose selection process for newer drugs, and design alternative strategies, the FDA has undertaken several initiatives in recent years to address these concerns. These actions include the launch of Project Optimus in 2021 and the issuance of draft guidance for industry on dose optimization of oncology drugs in 2023. Amid this evolving regulatory environment, the present manuscript reviews case studies for six different targeted cancer therapies, highlighting how dose-finding challenges have been managed to date by oncologists, sponsors, and regulators.

## 1. Introduction

Declining cancer mortality rates in the US have been attributed in part to breakthroughs in treatment, including the emergence of targeted therapies [1]. Targeted therapies constituted 82.8% of all FDA-approved oncology drugs between 1998 and 2022, and have altered the treatment paradigm for many cancers [2]. Despite the profound changes in clinical practice catalyzed by targeted anticancer therapies, parallel changes have not been observed in the drug development process for these drugs, with dose-finding methodology continuing to follow practices established for cytotoxic chemotherapy. In an effort to reform dose selection practices for cancer therapies, the FDA recently rolled out the Project Optimus initiative and published a draft guidance for industry on optimizing the dosage of drugs and biological products for the treatment of oncologic diseases [3,4].

Elucidating the dose to advance to registrational trials is a foundational step in the drug development process. In the 1994 International Conference on Harmonisation (ICH) E4 guidance on Dose-Response Information to Support Drug Registration, prospective, randomized clinical trials are recommended to assess dose response [5]. For indications where the condition is life-threatening, the guidance notes that greater levels of toxicity may be considered acceptable, and so higher doses are often selected in order to rapidly produce maximal therapeutic effect. However, the guidance warns that “this approach may lead to recommended doses that deprive some patients of the potential benefit of a drug by inducing toxicity that leads to cessation of therapy”, and that “the highest tolerated dose…will not always be the optimal dose” [5]. Although three decades have elapsed since the ICH E4 guidance for industry was published, these principles remain relevant to dose optimization of targeted therapies for oncologic indications today.

**Figure 1:**
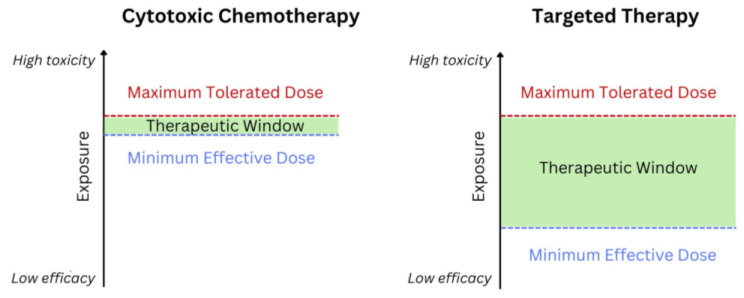
Therapeutic windows for cytotoxic chemotherapy vs. targeted therapy.

The conventional paradigm for dose selection of oncology drugs was originally developed for cytotoxic chemotherapy. Cytotoxic chemotherapeutics indiscriminately kill dividing malignant and non-malignant cells, resulting in significant off-target toxicity. The toxicity of these agents increased concurrently with efficacy, so the most efficacious dose was often the maximum tolerated dose (MTD). While this approach was suitable for the narrow therapeutic window, limited specificity, and short treatment duration of cytotoxic chemotherapy, contemporary targeted therapies have different properties (Figure 1). Targeted anticancer therapies are designed to hone in on cells with tumorigenic mutations specifically. Sufficient target inhibition may occur—and efficacious responses may be observed—at much lower doses than the MTD, creating flatter dose-response curves and a wider therapeutic window.

In addition, targeted therapies are generally administered for longer periods of time than conventional cytotoxic treatment modalities, and therefore tolerability is of greater importance. In spite of these differences in the characteristics of the oncology drugs under study, dose-finding practices have largely adhered to the historical precedent: an analysis of 116 new molecular entities approved by the FDA for oncology indications between 2010 and August 2021 found that nearly 40% used the MTD as the recommended dose in the prescribing information [6].

The ramifications of selecting a higher dose than is necessary are evident in the toxicity observed among patients. A survey conducted by the Patient-Centered Dosing Initiative found that among 1221 patients with metastatic breast cancer, 86% had experienced a significant treatment-related side effect, which led to 43% missing at least one treatment, and 20% being admitted to the emergency room or hospital [7]. Of those patients who subsequently had their dose reduced, 83% reported feeling better. Treatment-related toxicity need not result in hospital admission to be detrimental to patients’ health and well-being: chronic lower-grade symptomatic adverse events such as diarrhea can negatively impact patients’ quality of life, and be disruptive and distressing enough to prevent continuation of therapy. Missing doses can limit sustained target inhibition of the therapy, reducing its effectiveness and preventing patients from deriving the full benefit of the treatment.

## 2. Investigator and Sponsor-Initiated Dose Optimization in the Post-Marketing Setting

Oncologists in clinical practice are acutely aware of the challenges of treating patients with drugs approved at suboptimal doses, and have devised numerous strategies to manage dosing of cancer therapies in order to mitigate toxicities and facilitate keeping patients on treatment. For example, in a survey of 119 medical oncologists, 87% indicated that they had started patients on a lower than recommended dose [8]. Significantly, 85% of oncologists surveyed did not believe that the recommended dose was necessarily more effective than a lower dose. This belief may well be rooted in fact. One example is ibrutinib, a Bruton tyrosine kinase (BTK) targeting inhibitor that was granted accelerated approval by the FDA on 12 February 2014 for the treatment of patients with chronic lymphocytic leukemia (CLL) who have received at least one prior therapy, at a fixed dose of 420 mg daily [9]. The approval was based on 48 patients in the open-label, multi-center Phase 1b/2 PCYC-1102-CA study (NCT01105247) [10]. Although the objective response rate (ORR) (assessed using a modified version of the International Workshop on CLL Criteria by an Independent Review Committee) observed in the trial was 58.3% (95% confidence interval (CI): 43.2–72.4%), 42% of patients required a dose modification or interruption due to an adverse event [11]. No post-marketing requirements (PMRs) or commitments (PMCs) for dose optimization studies were issued [9]. Real-world data have shown that dose reductions, holds and discontinuations (common hallmarks of an intolerably high dose) are prevalent in patients receiving ibrutinib for CLL [12], with one study finding that 41% of patients with CLL discontinued treatment due to intolerance [13]. Evidence suggests that the 420 mg daily dose may not be required for efficacy: an early Phase 1 study found that full BTK occupancy was achieved at a dose of 2.5 mg/kg (equivalent to approximately 175 mg in an average adult weighing 70 kg) [14]. This observation was later confirmed by a second study, which demonstrated that a stepwise reduction from a daily dose of 420 mg to a daily dose of 280 mg or even 140 mg maintained >95% BTK occupancy [15]. While this data supports the potential for adoption of a lower dose, further investigation will be needed to confirm clinical efficacy.

Research indicates that these types of studies would be welcomed by healthcare practitioners. A survey of 367 oncologists found that nearly all (89%) were in favor of future trials to optimize cancer drug dosing [16]. However, pending completion of such studies (which may never be conducted by the sponsor for drugs already on the market, absent a regulatory mandate to do so), some oncologists have engaged in research of their own to investigate alternative dosing strategies. One example is that of pazopanib, a drug for which researchers took advantage of a known food effect in an attempt to improve tolerability (see Section 2.1).

### 2.1. Case Study 1

Pazopanib is a tyrosine kinase inhibitor approved by the FDA on 19 October 2009 for the treatment of patients with advanced renal cell carcinoma [17]. The approved dose was 800 mg orally once daily without food (at least 1 h before or 2 h after a meal) [18]. This dose was identified based on the Phase 1, 3 + 3 design VEG10003 trial (NCT00060151) [19]. According to the prescribing information, a significant food effect was observed with pazopanib: when taken with a high-fat meal, pazopanib exposure was approximately doubled (two-fold increase in area under the curve and maximum concentration). The drug’s approval was based on data from 435 patients in a randomized, double-blind, placebo-controlled multi-center study of pazopanib vs. placebo in patients with locally advanced and/or metastatic renal cell carcinoma who had received either one or no prior systemic cytokine-based therapy (NCT00334282) [20]. The median progression-free survival (PFS) was 9.2 months among patients treated with pazopanib, compared to a median PFS of 4.2 months among patients receiving placebo (hazard ratio (HR) 0.46; 95% CI: 0.34–0.62; *p* < 0.0000001) [18]. However, patients receiving pazopanib reported more Grade 3 and 4 adverse events and serious adverse events than patients receiving placebo [21]. Pazopanib-treated patients also required more dose modifications relative to placebo-treated patients, experiencing a greater incidence of both dose interruptions (35% vs. 10%) and reductions (25% vs. 4%) [21]. No PMRs or PMCs for dose optimization studies were issued at the time of approval [17]. The DIET study (NCT02138526) was an independent investigation conducted post-approval to determine if the known food effect could be leveraged to reduce gastrointestinal (GI) adverse events (which were a common reason for dose reductions in the pivotal trial) while maintaining comparable pazopanib exposure [22]. The study compared the bioavailability of the 800 mg dose of pazopanib administered in the fasted state (i.e., recommended dosing per the prescribing information) to a 600 mg dose taken with a continental breakfast, and found that the results for the two regimens were equivalent [22]. While there were no differences observed in GI adverse events, patients reported higher satisfaction with the 600 mg dose of pazopanib taken with food, and most patients indicated that they preferred this more convenient regimen (factors which could potentially lead to better adherence to treatment) [22]. The DIET study did not result in a change to the pazopanib prescribing information, and the impact of this study on real-world dosing of pazopanib is unknown.

Another example of a cancer drug that healthcare providers have endeavored to improve the tolerability of, and increase the duration of therapy with, is regorafenib. Section 2.2 describes how oncologists developed a dose escalation scheme for regorafenib, which successfully influenced real-world treatment patterns for this drug.

### 2.2. Case Study 2

Regorafenib, a multi-targeted kinase inhibitor, was granted regular approval by the FDA on 27 September 2012, for the treatment of patients with metastatic colorectal cancer who have been previously treated with fluoropyrimidine-, oxaliplatin- and irinotecan-based chemotherapy, an anti-vascular endothelial growth factor (VEGF) therapy, and, if KRAS wild type, an anti-epidermal growth factor receptor (EGFR) therapy [23]. The approved dose was 160 mg orally once daily for the first 21 days of each 28-day cycle, a dose identified as the MTD in the Phase 1 Study 11650, which used a 3 + 3 design [24,25]. The drug’s approval was supported by the Phase 3 CORRECT trial (NCT01103323), which demonstrated an overall survival benefit among patients treated with regorafenib compared to those treated with placebo (6.4 months in the regorafenib group vs. 5.0 months in the placebo group (HR 0.77; 95% CI: 0.64–0.94; *p* = 0.0052)) [26]. However, tolerability of the drug was poor, with treatment-related adverse events often requiring dose reductions or interruptions. In the CORRECT trial, 333 (67%) of 500 patients in the regorafenib group had adverse events leading to dose modifications, compared to 57 (23%) of 253 patients in the placebo group [27]. In total, Grade 3 or 4 treatment-related adverse events were reported in 270 (54%) patients treated with regorafenib, and serious adverse events were reported in 219 (44%) [27]. The most frequent treatment-related adverse events of Grade 3 or higher were hand-foot skin reaction (palmar-plantar erythrodysesthesia syndrome (PPES)), fatigue, diarrhea, hypertension, and rash. The majority of adverse events were reported in the first few 1–2 cycles of treatment with regorafenib [27].

**Figure 2:**
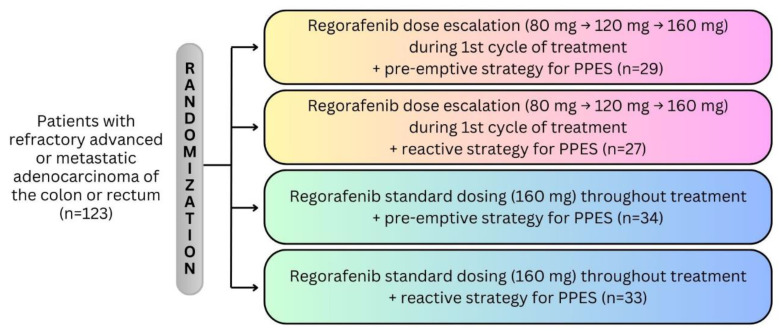
The randomized, parallel arm design of the regorafenib reDOS trial.

Since its approval, oncologists have undertaken numerous investigational strategies to improve the tolerability of regorafenib while maintaining its efficacy. One strategy involved titration to the approved dose, to mitigate adverse events commonly observed early in treatment. In the reDOS trial (NCT02368886), 123 patients were randomized to one of four regorafenib treatment groups: standard dosing plus preventive clobetasol cream (for PPES), dose escalation plus preventive clobetasol cream, standard dosing plus reactive preventive clobetasol cream, and dose escalation plus reactive preventive clobetasol cream [28] (Figure 2).

For patients in the dose escalation arms, dosing was 80 mg per day for the first week, followed by 120 mg per day the second week, and 160 mg per day the third week. Comparing the pooled dose escalation arms to the pooled standard dosing arms, a significantly greater proportion of the patients within the dose escalation arms completed two cycles of treatment and started Cycle 3 [28]. Progression-free survival was similar between the dose escalation group and the standard dosing group, however fewer patients in the dose escalation group experienced Grade 2–3 PPES compared to the standard-dose group. Patients in the dose escalation group also reported higher quality of life scores than patients in the standard dosing group for multiple items on the Brief Fatigue Inventory questionnaire at week 2 of treatment [28]. Taken together, this study provided preliminary evidence to suggest that a dose escalation strategy could help keep patients with metastatic colorectal cancer on regorafenib therapy longer, without compromising efficacy.

The reDOS study did not lead to a change in the regorafenib prescribing information, however, in 2018 the National Comprehensive Cancer Network (NCCN) guidelines for colon cancer referenced the published results and recommended the dose-escalation strategy as an appropriate alternative approach for regorafenib dosing [29]. A retrospective study of regorafenib dosing in the US which drew upon claims data found that, following inclusion of the dose-escalation strategy in the NCCN guidelines, the proportion of patients treated using the strategy more than doubled [30]. Importantly, more patients initiated a third treatment cycle, and the mean number of cycles per patient increased.

Although the burden of finding practical alternative dosing strategies often falls to oncologists in the clinic, sponsors may decide on their own to explore further dose optimization of an approved drug in the post-marketing setting (without impetus from regulatory authorities). One example is illustrated in the case of niraparib (see Section 2.3). Post-approval, the sponsor applied predictive modeling and analysis of existing data to demonstrate that a lower dose was safe and efficacious for a subset of patients who had experienced substantial toxicity in the pivotal trial. In this case, the prescribing information for the drug was updated to include revised dosing recommendations.

### 2.3. Case Study 3

Niraparib, a poly-ADP ribose polymerase 1–2 inhibitor, was granted regular approval by the FDA on 27 March 2017 for the maintenance treatment of adult patients with recurrent epithelial ovarian, fallopian tube, or primary peritoneal cancer who are in a complete or partial response to platinum-based chemotherapy, at a dose of 300 mg once daily [31]. This dose was identified as the MTD in the Phase 1 PN001 study, which employed an accelerated titration, 3 + 3 design (NCT00749502) [32]. The approval was supported by the Phase 3 randomized, double-blind, placebo-controlled NOVA trial (NCT01847274) [33]. In this trial, patients treated with niraparib had a significantly longer median duration of PFS than did those treated with placebo (21.0 vs. 5.5 months in the germline BReast CAncer gene (BRCA) mutation cohort (HR 0.27; 95% CI: 0.17–0.41), and 9.3 months vs. 3.9 months in the non-germline BRCA mutation cohort (HR 0.45; 95% CI: 0.34–0.61), *p* < 0.001 for both) [34]. Grade 3 or greater related adverse events were reported for 237 (64.6%), and serious adverse events were reported for 110 (30.0%) [34]. However, more than half of the patients receiving niraparib in the NOVA trial required dose modifications. A total of 253 of the 367 niraparib-treated patients (69%) had adverse events requiring dose reductions to 200 mg daily; 128 (35%) then had subsequent adverse events requiring a second dose reduction to 100 mg daily [35]. The majority of the dose reductions were due to cytopenias. No PMRs or PMCs for dose optimization studies were issued at the time of approval [31]. In the review package, the FDA reviewer states, “…in light of the compelling efficacy seen with niraparib therapy, it was determined that the proposed starting dose of 300 mg daily is the acceptable dose. Product labeling provides adequate guidance to clinicians on when and how to institute when dose reductions and interruptions, based upon toxicities, most of which are hematologic” [34].

After approval, the sponsor undertook a retrospective analysis using predictive modeling methods to identify factors associated with increased risk of developing Grade 3 thrombocytopenia within 30 days after the first dose of niraparib. The analysis found that baseline body weight <77 kg or baseline platelet count <150,000/μL were significant predictors for developing Grade 3 or greater thrombocytopenia [36]. Further analysis determined that PFS remained consistent among patients who were dose reduced to either 200 or 100 mg compared to those patients who were treated with 300 mg throughout the study [36]. The prescribing information for niraparib was subsequently changed in 2020 to include the following statement: “For patients weighing <77 kg (<170 lbs) OR with a platelet count <150,000/μL, the recommended dose is 200 mg taken orally once daily. For patients weighing ≥77 kg (≥170 lbs) AND a platelet count ≥150,000/μL, the recommended dose is 300 mg taken orally once daily” [37]. No additional studies were conducted to support this change in dosing recommendations.

## 3. FDA Strategies for Managing Dose Optimization in the Post-Marketing Setting

For cancer therapies approved at a dose that has not been optimized, the FDA may elect to mandate post-marketing studies in order to “repair the cracks” [38]. A Friends of Cancer Research (FOCR) analysis found that, of 132 novel oncology drug approvals between 2012 and 2022, 78 (59.1%) were issued PMRs to collect additional dosing information [39]. The study also ascertained that PMRs for studies intended to assess the safety and efficacy of lower doses have risen sharply, increasing from 22.2% of PMRs in the time period 2012–2015 to 55.6% in 2020–2022 [39]. The requirement to investigate a lower dose is often driven by toxicity and tolerability concerns. One recent example of a drug with PMRs issued to evaluate a lower dose is futibatinib (see Section 3.1). This case highlights a number of the recommendations spelled out in the FDA draft guidance on dose optimization, such as the randomized, parallel arm design, use of more than one dose, the longer time period of evaluation, characterization of exposure-response relationships (for both safety and efficacy) and dose-response relationships, as well as the inclusion of patient-reported outcomes [4].

### 3.1. Case Study 4

Futibatinib, a fibroblast growth factor receptor (FGFR) 1–4 inhibitor, was granted accelerated approval by the FDA on 30 September 2022 for the treatment of adult patients with previously treated, unresectable, locally advanced or metastatic intrahepatic cholangiocarcinoma harboring FGFR2 gene fusions or other rearrangements [40].

The approval was based on Study TAS-120-101 (NCT02052778), a Phase 1/2 multicenter, open-label, single-arm trial of futibatinib in patients with advanced solid tumors refractory to standard therapies and harboring FGF/FGFR alterations. In the Phase 1 part of the study, which employed a 3 + 3 design, the MTD was determined to be 20 mg once daily [41]. In the Phase 2 part of the study, 103 patients with advanced intrahepatic cholangiocarcinoma harboring FGFR2 gene fusions or other rearrangements received futibatinib at a dose of 20 mg once daily [42]. The ORR, as assessed by an independent review committee using Response Evaluation Criteria in Solid Tumors v.1.1, was 42% (95% CI: 32–52) and the median duration of response was 9.7 months (95% CI: 7.6–17.1) [43]. Grade ≥ 3 adverse reactions were reported for 77% of patients, and serious adverse reactions were reported for 39% of patients [44]. Dose modifications were frequently required for the management of toxicity, with dose interruptions reported for 66% of patients, and dose reductions reported for 58% of patients [44]. In the FDA approval package, reviewers noted: “In FDA’s analysis, the recommended dosage of futibatinib has not been adequately justified. The dosage was selected on the MTD of the first-in-human study and has not been optimized. Exploratory analyses suggest a negative exposure-response and a positive exposure-toxicity relationship…” [44]. The FDA issued the following PMR (4345-1) under accelerated approval: “Conduct a randomized clinical trial comparing dosages of futibatinib 16 mg and 20 mg once daily to verify and describe the clinical benefit of futibatinib in patients with advanced or metastatic cholangiocarcinoma harboring an FGFR2 gene fusion or other rearrangement. The overall response rate and duration of response should be assessed by a blinded independent review. The study should also evaluate other clinical outcomes that denote clinical benefit, such as patient reported outcomes. This study should enroll a minimum of 120 patients and all responders should have a minimum of 6 months from the date of initial response (or until disease progression, whichever comes first). Ensure that racial and ethnic minorities are adequately represented in the trial population, at a minimum, proportional to the prevalence of FGFR2 alterations in these subgroups in the US population” [40]. A second PMR (4345-4) was issued under section 505(o)(3) of the Federal Food, Drug, and Cosmetic Act, to better characterize safety: “Conduct a randomized study that compares the recommended dosage of 20 mg daily to a lower dosage (e.g., 16 mg) to provide a comparative analysis of dose-and exposure-response relationships for safety including further characterization of the rates of Grade ≥3 adverse reactions, Grade ≥3 hyperphosphatemia, serious adverse reactions, and dose reductions, interruptions, and discontinuations due to adverse reactions. Incorporate systematically assessed patient-reported outcome assessments to evaluate tolerability. Core outcomes should include patient-reported symptomatic adverse event data, overall side effect bother, physical function, and role function. The study should also provide a comparative analysis of dose-and exposure-response relationships for efficacy, including overall response rate and duration of response” [40].

A study comparing dosages of futibatinib 16 mg and 20 mg once daily (Study of Futibatinib in Patients with Advanced Cholangiocarcinoma With FGFR2 Fusion or Rearrangement, FOENIX-CCA4 (NCT05727176)) is currently recruiting patients.

While PMRs and PMCs represent one stop-gap measure at the FDA’s disposal to deal with inadequately dose-optimized drugs, it is a less-than-ideal workaround. Dose optimization studies in the post-marketing setting are resource and time-intensive, and can be difficult to enroll. The FOCR study determined that PMRs to evaluate dosing variations were given a median 4.5 years for completion (from drug approval to final study report due date) [39]. An FDA analysis of 138 novel cancer therapies approved between 2010 and 2022 found that completed dose optimization PMRs took a median of 6 years [38]. This extended period of time between approval and the answers to outstanding questions regarding dosing can translate directly into a greater burden for patients and providers striving to use the therapy. Furthermore, completed post-marketing trials or analyses of alternative dosages did not often lead to in changes to the prescribing information: the FDA analysis found that only five drugs had dosage changes resulting from 11 fulfilled PMRs during the time period analyzed [38]. Based on this data, sponsors may surmise that postponing dose optimization to the post-marketing period is worth the risk.

In some instances, concerns with dose may be significant enough that PMRs or PMCs will not be sufficient recourse to address them. In the case of melphalan flufenamide, “lack of an appropriate dose” was one of the issues that ultimately led to its withdrawal from the market (see Section 3.2).

### 3.2. Case Study 5

Melphalan flufenamide, a peptide-conjugated alkylating agent, was granted accelerated approval by the FDA on 26 February 2021 for the treatment (in combination with dexamethasone) of adult patients with relapsed or refractory multiple myeloma who have received at least 4 prior lines of therapy and whose disease is refractory to at least one proteasome inhibitor, one immunomodulatory agent, and one CD38-directed monoclonal antibody [45]. The approval was based on data from the Phase 2 HORIZON study (NCT02963493), a multicenter, open-label, single-arm trial [46]. In the HORIZON study, 157 patients were treated with 40 mg melphalan flufenamide, a dose identified as the MTD in the Phase 1, 3 + 3 design O-12-M1 study (NCT01897714) [47]. Among the 97 patients in the HORIZON study who had received four or more prior lines of therapies and were refractory to at least one proteasome inhibitor, at least one immunomodulatory agent and a CD38-directed monoclonal antibody, the ORR (assessed by investigators, using International Myeloma Working Group Criteria) was 23.7% (95% CI: 15.7–33.4) and the median duration of response was 4.2 months (95% CI: 3.2–7.6) [48]. Grade 3 or 4 adverse events were reported in 95.5% of patients, and serious adverse events were reported in 56.1% of patients in the HORIZON trial [49]. Adverse events leading to drug discontinuation were reported in 28.0% of patients, while 31.2% of patients had dose reductions and 65% had dose delays due to adverse events [49]. These observations led to FDA reviewers concluding that: “The safety concerns and the toxicity observed indicate that the flat dose of 40 mg is not optimized to support a favorable benefit-risk profile” [49]. Notably, pharmacokinetic data were not collected in the HORIZON trial, and thus no exposure-response analyses were conducted [49]. At the time of approval, PMRs were issued under 505(o) requesting further characterization of the exposure, safety and efficacy of melphalan flufenamide, in both the low body weight patient population and in US racial and ethnic minorities [45].

At the time of accelerated approval, the confirmatory multicenter, open-label randomized, controlled Phase 3 OCEAN trial (NCT03151811) evaluating melphalan flufenamide with dexamethasone compared to pomalidomide and dexamethasone in patients with relapsed or refractory multiple myeloma who had received 2–4 prior lines of therapy and were refractory to lenalidomide in the last line of therapy, was underway. In the OCEAN trial, 495 patients were randomized 1:1 to receive either melphalan flufenamide (40 mg) with dexamethasone or pomalidomide and dexamethasone [50]. An increased rate of death was observed among patients melphalan flufenamide with dexamethasone (117/248; 47.6%) compared to those receiving pomalidomide and dexamethasone (108/249; 43.4%) [49]. The median overall survival was 19.7 months among melphalan flufenamide treated patient vs. 25.0 months among pomalidomide treated patients (HR 1.104; 95% CI: 0.846–1.441) [49]. The sponsor subsequently voluntarily withdrew the drug from the US market on 22 October 2021 [51]. However, the sponsor later rescinded the withdrawal on 21 January 2022 [52]. On 22 September 2022, an Oncologic Drugs Advisory Committee meeting was held to review the benefit/risk profile of melphalan flufenamide for the indicated patient population. In addition to the potential detriment in overall survival observed in the OCEAN trial, other issues for discussion included failure to demonstrate a PFS benefit, and lack of an appropriate dose [49].

The Committee voted 14 to 2 that the benefit/risk profile of melphalan flufenamide was not favorable for the indicated patient population [53]. On 7 July 2023, the FDA notified the sponsor of their intention to withdraw approval of melphalan flufenamide [54], and on 23 February 2024, the FDA finalized this decision [55].

Bringing a new oncology drug to market is already a high-risk venture, with likelihood of regulatory approval only 5.3% (based on candidates in development for oncology indications between 2011 and 2020) and cost of development estimated at up to USD 4.5 billion (based on drugs approved by the FDA between 2009 and 2018) [56,57]. As stated by Richard Pazdur, the director of the FDA Oncology Center of Excellence, at a 2022 FDA-American Society for Clinical Oncology (ASCO) workshop on dose optimization of oncology drugs, “…developing a drug development program without having a thorough understanding of drug dose is akin to the proverbial building a house on quicksand” [58]. The pressure to deliver new therapies to patients in need as quickly as possible is undeniable, and the use of expedited programs like accelerated approval and breakthrough therapy may result in increased clinical development timeline compression [59]. However, rushing through or deferring dose exploration could jeopardize an entire program. The saga of melphalan flufenamide provides a compelling rationale to prioritize dose optimization earlier in drug development. A proactive, intentional approach from the start could obviate the need to resort to (often unsatisfactory) post-hoc fixes, or prevent the late realization that a drug development program should have been terminated much earlier.

## 4. Shifting Dose Optimization into the Pre-Marketing Setting

As outlined in the FDA’s 2023 draft guidance and in FDA-authored scientific publications on the subject, dose-finding should go beyond simply identifying the MTD [60,61]. Determination of the MTD relies on acute toxicity. For a 3 + 3 study design (the most commonly employed Phase 1 dose escalation design for cancer drugs), MTD is defined as the dose level at which less than 33% of patients experience a dose-limiting toxicity (DLT) in the first cycle of treatment (typically 28 days). The 3 + 3 design is the first choice for many investigators because the algorithm is straightforward, the endpoint of DLTs is binary, and the period of assessment is short. However, the design also has its drawbacks, including poor statistical properties compared to other designs [62]. A recent analysis of published data from 22 trials of oncologic drugs that utilized the 3 + 3 design found that the recommended MTD using the Bayesian optimal interval design would have matched the published MTD only 64% of the time, and using the continual reassessment method, just 55% of the time [63]. Another limitation of the 3 + 3 design is that serious toxicities may not emerge within the single cycle timeframe evaluated, owing to the different safety profiles of targeted therapies and other newer agents have compared to their predecessor cytotoxic chemotherapies [64]. Consequently, the MTD may never be identified: an analysis of oncology drugs approved by the FDA between 2010 and 2021 found that for more than half, no MTD was determined [6]. Despite the shortcomings of this method of dose-finding for modern cancer therapeutics, approximately two-thirds of first-in-human trials for small molecule oncology drugs approved by the FDA between 2011 and 2019 used the 3 + 3 design [65]. The FDA has encouraged innovation in trial designs for dose optimization and the need to incorporate other elements beyond acute toxicity, such as efficacy, pharmacokinetics, patient-reported outcomes, and non-clinical data (e.g., target engagement or receptor occupancy studies) [66]. A plethora of publications presenting new models and variations on existing designs have been released in the past year [67,68,69,70]. One example of a new oncology drug in development that utilized an alternate method for dose finding is zorifertinib, employing the Simon 2-stage minimax design (see Section Case Study 6). Although this model-assisted study design is not new, it exemplifies a strategy that does not require a large number of patients to identify a safe and efficacious dose.

### Case Study 6

Zorifertinib (AZD3759) is an investigational EGFR tyrosine kinase inhibitor in development for the treatment of patients with EGFR-mutated non-small cell lung cancer (NSCLC) and central nervous system metastases. In the open-label, Phase 2, adaptive umbrella trial CTONG1702 (NCT03574402), one arm evaluated the efficacy and safety of 200 and 300 mg doses of zorifertinib monotherapy (administered twice daily (BID)) in previously untreated patients with EGFR-sensitive mutations and locally advanced or metastatic NSCLC (and brain or leptomeningeal metastasis) [71]. These two doses were identified in a Phase 1 dose escalation and expansion trial (NCT02228369) which adopted a Bayesian adaptive design [72,73].

**Figure 3:**
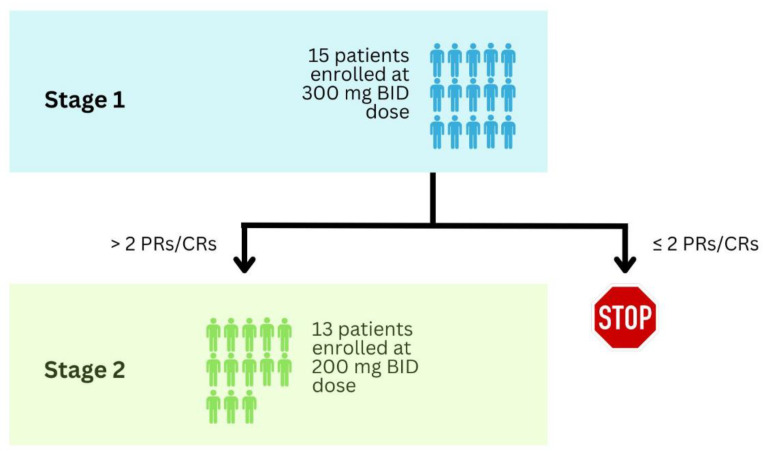
The Simon 2-stage minimax design of the CTONG1702 trial zorifertinib arm.

The study arm evaluating zorifertinib used Simon’s 2-stage minimax design [74]. This design primarily functions to minimize the maximum sample size. The assumptions used in the CTONG1702 zorifertinib arm included an unexpected ORR rate of 15%, an expected ORR rate of 35%, a type I error rate of 5%, and 80% power [71]. Factoring in a 10% dropout rate, 31 patients would need to be enrolled in the treatment cohorts. The study schema (Figure 3) was as follows: 15 patients would enroll in Stage 1, and if more than two patients achieved a partial response (PR) or complete response (CR), then Stage 2 would be opened for enrollment to 13 patients. If two or fewer patients achieved PR or CR, the study would be terminated. A total of 28 patients would be enrolled in the zorifertinib arm [71].

In Stage 1, 15 patients were enrolled and treated with 300 mg BID [71]. An interim analysis indicated that eight of the 15 experienced PRs. In Stage 2, another 15 patients were enrolled and treated with 200 mg BID. At the time of data cut-off, there were 12 responders in the 200 mg group, and nine responders in the 300 mg group. The ORR was 80% and 60% in the 200 and 300 mg cohorts, respectively, and median PFS was 15.8 and 10.7 months in the 200 and 300 mg cohorts, respectively [71]. Grade 3–4 treatment-related adverse events occurred in 60% of patients in the 200 mg group, vs. 87% of patients in the 300 mg group. The 200 mg cohort also had lower rates of dose interruption, dose reduction, and permanent treatment discontinuation. The results of this study informed the selection of the 200 mg BID dose for the Phase 2/3 EVEREST study of zorifertinib [71].

In January 2023 the sponsor announced that, following the successful completion of the EVEREST study, the New Drug Application for zorifertinib had been submitted to the Center for Drug Evaluation of the National Medical Products Administration in China [75]. Top-line results from the EVEREST study released in June 2023 indicated that while efficacy was superior among patients treated with zorifertinib 200 mg BID compared to those treated with an active control (first generation EGFR tyrosine kinase inhibitors gefitinib 250 mg or erlotinib 150 mg once daily), Grade ≥3 treatment-related adverse events were reported in 65.9% of patients in the zorifertinib group vs. 18.3% of patients in the control group [76]. This striking difference in toxicity suggests that even the 200 mg BID zorifertinib dose may have been too high.

One shortcoming of the zorifertinib arm of the CTONG1702 trial was that no pharmacokinetic samples were collected during the study, and so no further exposure response analyses could be conducted beyond those performed in Phase 1 [71]. This is problematic, as a clear understanding of exposure-response is “at the heart of any determination of the safety and effectiveness of drugs”, according to the FDA guidance on exposure-response relationships [77]. Progress is needed across the industry in this integral component of cancer drug development: a study of oncology drugs first approved by the FDA between 2010 and August 2021 found that no further dose-ranging or refinement occurred after Phase 1 dose escalation for approximately 80% of the therapies [6].

## 5. Recommendations from the FDA Guidance on Dose Optimization of Oncology Drugs

The FDA draft guidance document on optimizing the dosage of drugs for the treatment of oncologic diseases centers the concept that dose-finding is not simply a toxicity question to answer in Phase 1. Instead, sponsors should draw from all available information (including non-clinical data from animal and in vitro/in vivo studies; clinical safety, tolerability, and efficacy data; pharmacokinetics, pharmacodynamics and exposure-response data; patient-reported outcomes; as well as data related to biomarkers and tumor biopsies if applicable) to select a dose to carry forward into registrational trials.

The guidance recommends that Phase 1 studies establish a dose range (rather than a single dose) for further evaluation in Phase 2 [4]. A randomized, parallel-arm design to investigate two or more active doses is presented as a suitable approach to Phase 2 dose optimization studies, but other designs (including seamless Phase 1–2 designs with expansion cohorts, or novel designs) are also feasible. Adaptive features incorporating an interim analysis and pre-specified stopping rules (for efficacy/safety) are an efficient approach. The specific doses for the Phase 2 trial should be within the range identified in the Phase 1 study, but pharmacologically distinct based on available data. Modeling or simulation may aid in defining the doses for study. The Phase 2 trial should be of sufficient size to assess safety, tolerability and activity for each dose; however, it does not need to be powered for safety or efficacy outcomes. Assessments in the Phase 2 trial should include dose interruptions, dose reductions, and drug discontinuation due to treatment-related adverse events. Evaluating patient-reported outcomes could also serve to complement safety and tolerability data. To support the exploration of exposure-response relationships and population pharmacokinetics, a pharmacokinetic sampling and analysis plan should be pre-specified and provided to FDA for review. For some therapies, a plan for collection and analysis of pharmacogenomic or pharmacodynamic data may also be appropriate [4].

The guidance leaves some open questions, for example, it does not elaborate on how pharmacologically distinct the doses selected for Phase 2 dose optimization study need to be, or how large is large enough for a Phase 2 dose optimization study to sufficiently assess safety, tolerability and activity for the individual doses. However, it is understood that the unique properties of each targeted therapy require that dose optimization for each drug be considered on a case-by-case basis. While FDA guidance for industry is helpful, in reality there cannot be a set of “one-size-fits-all” instructions that will be appropriate for every targeted therapy. In addition, the optimal dose may differ depending on the indication and patient population, and therefore the dose optimization process may need to take place multiple times throughout a drug’s life cycle. For example, the kinase inhibitor dasatinib was approved for chronic phase Philadelphia chromosome-positive (Ph+) chronic myeloid leukemia (CML) in adults at a dose of 100 mg once daily, but for accelerated phase CML, myeloid or lymphoid blast phase CML, or Ph+ acute lymphoblastic leukemia in adults, the approved dose is 140 mg once daily [78]. Dosing for pediatric patients is based on body weight [78]. Finally, the optimal dose for an individual patient may not be the same as the approved dose (even when the approved dose is justified based on adequate dose optimization studies). For these patients, therapeutic drug monitoring can be an effective tool to improve efficacy while minimizing toxicity of targeted therapies [79]. Additionally, a personalized approach to finding the right dose, tailored on a patient-by-patient basis, is a practice supported by both patients and physicians [7,8]. Patients with cancer can exhibit great variability in exposure to anticancer agents, influenced by factors like age, body weight, renal and hepatic impairment [80]. These factors may change over time for an individual patient, and so continual reassessment and communication between the oncologist and the patient may be necessary to arrive at the most appropriate dose for that particular patient at that particular time.

## 6. Conclusions and Future Directions

The advent of precision oncology and the emergence of targeted therapies has improved the long-term prognosis for many patients with cancer. Despite the transformational changes that have revolutionized cancer treatment over the past several decades, the prevailing paradigm for dose-finding among cancer drugs has not kept pace with the advances. In the case studies of approved targeted anticancer therapies presented in this review, five of five used a 3 + 3 dose escalation Phase 1 study design, and four of five selected the MTD as the recommended Phase 2 dose (Table 1).

However, in light of growing recognition among patients, providers, regulators and the scientific community as a whole that “more is not always better”, change appears imminent [81]. Regulatory initiatives like Project Optimus and the issuance of draft guidance, aimed at reconfiguring dose-finding practices during drug development and prioritizing robust dose exploration in the pre-approval stage, have altered the tenor of the conversation. Dose optimization can no longer be viewed as an afterthought or a “nice to have”, but rather an essential element of oncology drug development. Additionally, recent investment in studies to support dose optimization and address evidence gaps have marked an increased interest in understanding benefit–risk trade-offs and identifying practical options for dosing oncology drugs in the real world. In the past year, the FDA’s Centers of Excellence in Regulatory Science and Innovation program funded the University of North Carolina (UNC) project “Using the UNC Clinical Data Warehouse to Evaluate the Benefit/Risk Ratio and Optimal Doses of Molecularly Targeted Therapies and Novel Biologics in Real World Patients”) and the Patient-Centered Outcomes Research Institute funded ASCO’s study of dosing strategies for oral CDK4/6 inhibitors in older adults living with metastatic breast cancer [82,83]. To further encourage refinement of dosing for approved targeted therapies, future initiatives might include legislation to provide incentives, such as an additional period of market exclusivity, for sponsors of approved drugs to conduct dose optimization studies post-marketing. Taken together, efforts to reshape approaches to dose-finding for anticancer therapies pre-approval, along with continued investigation to improve dosing strategies for approved drugs, will provide the best chance for patients to reap the benefits of these agents while minimizing avoidable toxicity.

## Figures and Tables

**Table 1:** Summary of case studies of approved targeted anticancer therapies.

Drug	Approved Dose	Was the Approved Dose the MTD (or Maximum Studied Dose) in the Phase 1 Dose Escalation Study?	Design of Phase 1 Dose Escalation Study	Was Further Dose Optimization Undertaken (beyond the Phase 1 Dose Escalation Study) Prior to Approval?	Dose Investigated Post-Marketing	Impact of Post-Marketing Dose Exploration
Pazopanib	800 mg without food	No	3+3	No	600 mg with food	None known
Regorafenib	160 mg	Yes	3+3	No	Dose escalation from 80 mg to 120 mg to 160 mg over the first cycle	Change to NCCN guidelines
Niraparib	300 mg	Yes	Accelerated titration, 3+3	No	200 mg in a subset of patients	Change to prescribing information
Futibatinib	20 mg	Yes	3+3	No	16 mg	N/A, study is still ongoing
Melphanan flufenamide	40 mg	Yes	3+3	No	N/A (drug withdrawn from market)	N/A

MTD = maximum tolerated dose; N/A = not applicable; NCCN = National Comprehensive Cancer Network.
